# Path Model Analysis of the Effects of Perceived Formalism, and Fear of COVID-19 on Police Officers’ PTSD and Insomnia

**DOI:** 10.3390/bs13100867

**Published:** 2023-10-23

**Authors:** Frank Fu-Yuan Huang, Hsiang-Te Liu

**Affiliations:** 1 Department of Criminal Justice, Ming Chuan University, Taoyuan City 333, Taiwan; 2 Department of Public Affairs and Administration, Ming Chuan University, Taoyuan City 333, Taiwan

**Keywords:** job stress, fear of COVID-19, job burnout, PTSD, insomnia, perceived formalism

## Abstract

This study examines whether law enforcement officers’ fear of COVID-19, job burnout, and job stress have increased their PTSD and insomnia during the epidemic. This article introduces the perceived formalism of police agencies into the causal model to explore whether perceived formalism increases the job burnout and job stress of police officers. The formalism of administrative agencies is rarely included in epidemic research. This study collected 306 Taiwanese police officers as research subjects. We used confirmatory factor analysis and SEM for hypothesis testing. The study found that perceived formalism positively affects the job burnout and job stress of police officers. Job stress, fear of COVID-19, and job burnout positively affect PTSD and insomnia.

## 1. Introduction

An important contribution of this study is incorporating the formalism of police administration into the study of law enforcement during a pandemic. Nearly half a century after Riggs proposed Eastern administrative formalism, empirical studies remain virtually non-existent [1]. This study can fill the gap in past research on formalism. Formalism is defined as a difference between what is formally prescribed and what is actually practiced [2]. As for the emergence of formalism, Riggs argued that it came about as a result of traditional societies invoking Western administrative models [3]. Developing countries are considered to follow a “prismatic model”, which is different from Western industrial and traditional societies [3,4]. The differences in administrative ecology cause systems that work in the West to have other effects when they are introduced to developing countries.

Institutions practicing formalism attach great importance to formal procedures, but not to performance or outcomes [5]. This is where the inconsistency between actual policy performance and purported implementation outcomes occurs. Managerial confusion arises from the inconsistency between formal and substantive authorities [5]. Milne pointed out that civil servants in a formalist state are full of insecurities [5], which comes from absolute authority, lack of empowerment, and lack of communication. Their insecurity, in turn, result in indifference and mistrust. The lack of effective resources and techniques is one of the characteristics of the formalist state [5]. When faced with new pandemic control situations, with every country suffering inadequate equipment and resources, police officers are more likely to feel stressed and burned out. The personal and organizational goals of civil servants are often inconsistent, even divergent [5]. When police officers‘ personal goals and organizational goals do not match, they are prone to feel stress and burnout, which may eventually lead to PTSD and insomnia.

PTSD is considered to be the experience of psychological trauma that causes disruption of life for more than a month. The COVID-19 pandemic has threatened the lives and work of the general population [6]. Social isolation has prevented schools, government agencies, and stores from functioning normally. The COVID-19 pandemic is considered a traumatic event that triggered PTSD [6]. Past Ebola outbreaks in Africa have also caused PTSD in first responders [7]. A study by Sun, et al. noted that people who had more contact with COVID-19 patients were more likely to develop PTSD [8]. Another study similarly demonstrated a vulnerability to PTSD from exposure to COVID-19 [9].

PTSD is recognized as a psychological disorder resulting from an individual’s exposure to natural disasters and diseases [10]. Exposure to deaths and illnesses caused by such diseases creates post-traumatic stress [11]. The uncertainty and unpredictability generated by the COVID-19 outbreak tend to produce fear and nervousness [12]. Fear of being infected is recognized as a cause of PTSD. Police officers who are responsible for law enforcement on the front line during the pandemic are prone to develop PTSD when they are exposed to death, sickness, and pain. Police work is often characterized by shifts without enough rest [13]; the frequent exposure to on-duty violence, and the risk of viral infections multiplies the job’s stress. Numerous studies have suggested that infectious diseases can cause anxiety [14], which in turn can lead to insomnia.

Fear of COVID-19 has been linked to insomnia [15]. Fear of COVID-19 causes neurobiological alterations [16]. These alterations cause sleep disorders and psychological problems [17]. Coronaphobia is the persistent fear of exposure to COVID-19. Fear of COVID-19 causes tremors, shortness of breath, and insomnia (Arora, et al., 2020) [18]. Fear of COVID-19 affects the mental health of frontline personnel [19].

Job burnout is a combination of symptoms caused by chronic, interpersonal, and work-related stress. Burnout symptoms include exhaustion, cynicism, and lack of self-efficacy [20]. Some scholars have also attributed job burnout to exhaustion and disengagement [21]. Exhaustion is considered to be physical, cognitive, and emotional depletion as a result of job demands, while disengagement is a negative attitude toward work and a desire to stay away from the job. Job burnout causes turnover and reduces performance [22].

COVID-19 poses a high mental health risk to frontline personnel [23]. Fear is seen as an emotional response by the average person during a pandemic [24,25]. Fear reduces self-efficacy and causes one to cope with pandemic anxiety via avoidance strategies [25]. Pandemic fear comes from the fear of infection of oneself and family members [26].

Sources of work stress for police officers include occupational and organizational sources. Police officers have been frontline workers during the pandemic. Police work has many norms, procedures, and rotations that are sources of stress [27]. As a result, anxiety, neuroticism, and post-traumatic stress disorder (PTSD) are always more severe in police officers than in those with general occupations [28]. Police work is considered a stressful job, and requires facing danger and uncertainty [29,30]. Police officers’ work stress, psychological exhaustion, and PTSD are recurrent problems [29,31].

## 2. Literature Review and Hypotheses Development

Law enforcement officers are exposed to many traumatic, stressful, and fatal events. Police officers’ highly stressful work environments cause them to develop physical and psychological problems. A study in the United Kingdom found that police officers are more prone to PTSD than the general population [32]. Police officers often encounter problems with mental illness and therefore are more prone to psychological stress than the general population [33]. Police officers who have been frontline workers during COVID-19 experience a lot of work stress and burnout.

According to the conservation of resources theory, individuals develop stress due to a loss of resources [34]. When police officers lose social support due to the pandemic, they are prone to PTSD. During periods of social isolation, police officers are also unable to see their family members and close friends. Social support is interrupted. Occupational stressors in police work include shift rotation, risk of accidental injury, traumatic events, and fear of excessive use of force. Organizational stress includes conflicts with co-workers, lack of resources, heavy workload, and large amounts of tasks. Physical and mental health are affected when work stress is high [35]. Anxiety, depression, and PTSD in police officers due to emotional exhaustion have been confirmed [36]. Many other studies have also confirmed that police job stress positively affects PTSD [37].

**H1:**  *Job stress positively influences PTSD (post-traumatic stress disorder).*

During COVID-19, individuals have been prone to anxiety, anger, and stress [38]. Fear of COVID-19 also predisposes individuals to PTSD. Fear is considered to be a fear of something specific, such as the spread of a virus [39]. Anxiety, on the other hand, is a fear of a wide range of unspecified targets, such as the COVID-19 outbreak as a whole. Fear, anxiety, and stress can all further contribute to PTSD. Anxiety and stress can further trigger PTSD [39].

Individuals are susceptible to stress, anxiety, pressure, fear, and PTSD when the number of confirmed outbreak cases and deaths from a pandemic increases rapidly [19,40]. In fact, these symptoms occurred during the Middle East respiratory syndrome (MERS) and severe acute respiratory syndrome (SARS) pandemics [41]. During the beginning of the outbreaks, there was stress and worry due to the unknown route of transmission and the lack of effective vaccines and medications.

Past research has confirmed that individuals feel anxious and fearful when faced with life’s vulnerabilities [8,42]. Infectious disease outbreaks are thought to cause post-traumatic stress disorder (PTSD), and Liu, et al. showed that COVID-19 has been shown to cause a significant proportion of PTSD [42]. Prior experience with SARS showed that 13.3% of males and 18% of females suffered from PTSD and developed stress, sleep disorders, sleep apnea, and anxiety [8]. PTSD is also associated with stress, sleep disorders, and alcoholism [43].

Past SARS research has also found that work with high exposure to infectious diseases predisposes one to PTSD [44]. PTSD can occur due to a loss of loved ones during a pandemic [45]. The highly infectious and lethal nature of COVID-19 in its early stages of development increased the occurrence of PTSD. Outbreak monitoring, travel restrictions, social distancing policies, and isolation all predisposed people to develop PTSD and psychological problems [38].

Some jobs are highly exposed to the risk of COVID-19 infection because of their work responsibilities. For example, construction site workers, healthcare workers, firefighters, and police officers were unable to work online during the outbreak. Frontline healthcare workers were found to be under high stress during the outbreak [46]. Previous studies of the SARS pandemic also found that frontline workers were prone to developing PTSD [47]. A survey in Hong Kong found that post-SARS frontline workers had higher levels of fear, anxiety, and PTSD than the general population [48].

COVID-19 has produced 2–3 years of high levels of stress, causing the general population to experience higher levels of fear [49]. The longer the traumatic experience is, the more severe the PTSD is. COVID-19 even caused cumulative traumatic stress during the outbreak [50]. Stresses brought on by the COVID-19 pandemic have included fear of infection, fear of death, financial hardship, and disruption of life [51]. Many of these concerns have resulted in PTSD, fear, and terror [52].

**H2:**  *Fear of COVID-19 positively influences PTSD.*

Many frontline disaster workers experience traumatic events [53]. Many frontline workers do not have the necessary training to work with traumatized individuals, which leads to job burnout. Job burnout is characterized by emotional exhaustion, depersonalization, and diminished feelings of personal accomplishment [54].

Job burnout creates frustration, anger, and exhaustion, further contributing to PTSD [55]. The relationship between job burnout and PTSD has been linked in the past. Symptoms of PTSD in firefighters include avoidance and mood changes [56]. Studies in the United States have found that police officers suffer from PTSD at a rate 10 times higher than that of the general population. Studies in Poland found that half of all police officers have experienced traumatic events, and that job burnout among police officers is higher as a result [57].

Experiencing trauma is an easy way to develop job burnout, which in turn increases PTSD. Viewed through a job demand model, job burnout occurs when individuals are faced with many job demands and insufficient job resources [58]. Viewed through the conservation of resources model, many stressors consume job resources and contribute to emotional exhaustion [59].

PTSD is caused by high levels of job burnout in many occupations. Experiencing negative emotions after a traumatic event and constantly thinking about the traumatic event can easily result in the development of PTSD [60]. Police officers are prone to developing PTSD by continually thinking negatively about a traumatic event [61]. Ruminating is a dysfunctional cognitive coping strategy that may cause PTSD to persist longer [62].

**H3:**  *Job burnout positively influences PTSD.*

Insomnia can cause fatigue, lack of energy, difficulty concentrating, decreased memory, and emotional instability [63]. Previous research has indicated that insomnia is an underestimated and underdiagnosed symptom [63]. Taylor also pointed out that insomnia is not always recognized as a serious health problem [64]. Anxiety and depression may be the cause of insomnia [65]. Insomnia, in turn, contributes further to depression and anxiety [64].

Insomnia induced by stressful events can be intermittent, recurrent, or chronic [63]. Insomnia has persisted during the pandemic, even after social distancing policies have ceased [63]. Uncertainty, worry, and anxiety associated with the pandemic have enhanced insomnia problems [66]. Pillai, et al. also found that prolonged stress and frequency of occurrence increase the risk of developing insomnia [67]. The frontline work that police officers are burdened with is bound to be quite stressful; this was especially true in the initial period, when COVID-19′s route of transmission remained unclear.

Pandemic-induced life interruptions, including telecommuting and lockdowns, have also been causes of insomnia [68]. Frontline workers have been overloaded with work; isolated from their families, relatives and friends; lacked adequate support; and even discriminated against [46]. High levels of stress during the pandemic have made insomnia, fatigue, and exhaustion frequent. Insomnia is considered one of the most important symptoms of stress. Exhaustion is an important characteristic of insomnia [69].

**H4:**  *Job stress positively influences insomnia.*

Fear of COVID-19 increases the risk of developing mental illnesses, including anxiety and insomnia [19]. Fear of COVID-19 is a psychological ‘coronaphobia’ regarding exposure to the virus. Physical symptoms include palpitations, tremors, shortness of breath, loss of appetite, and insomnia, all of which can affect an individual’s quality of life [18].

Insomnia has been recognized as a relatively serious problem during the pandemic [70]. Insomnia is a chronic problem consisting of difficulty in falling asleep, or a tendency to wake up too early. Past studies have found that personal anxiety and depression can affect sleep quality [71]. The fear of COVID-19, which is a highly life-threatening condition, can easily lead to problems with insomnia [72]. Fear of COVID-19 can cause negative psychological problems for an individual. Police officers dealing with outbreaks on the front line are bound to have a higher fear of COVID-19. Decreased personal contact during the implementation of lockdown policies decreased interactions with family and friends; fear of infection, work, and psychological stress has contributed to the development of fear of COVID-19 [38].

**H5:**  *Fear of COVID-19 positively influences insomnia.*

During the pandemic, frontline workers have experienced symptoms of stress, insomnia, and job burnout [73]. The rapid development of the pandemic, the sudden increase in workloads for frontline workers, and the lack of anti-pandemic equipment and clear anti-pandemic policies tended to make police officers have insomnia due to job burnout. Police officers have been faced with lockdowns and social distancing policies, and the increased workloads and shift changes have made them feel stressed and burned out. Job burnout does indeed tend to contribute to the development of PTSD [74].

High workloads and low social support are all causes of job burnout. Longer work hours and shift changes make police officers susceptible to job burnout. Past studies have indicated that job burnout is recognized as a cause of insomnia, resulting in poor sleep quality [75]. Police officers already have a demanding job, and the many uncertainties and policy changes they have faced during the pandemic make them prone to insomnia due to job burnout.

**H6:**  *Job burnout positively influences insomnia.*

Senior management with a high degree of formalism does not delegate authority to subordinates, and subordinates are also not adequately trained [31]. The COVID-19 outbreak was unprecedented, and general police forces lacked training in how to deal with it. A lack of training, experience, and resources for pandemic preparedness can lead to stress and burnout. Formalist organizations are full of insecurities, a lack of authorization, over-emphasis on regulations and documents, and a lack of communication [31]. The lack of vaccines and protective equipment during the COVID-19 outbreak made frontline police officers susceptible to work stress and burnout when they were not authorized to do their jobs and when communication was lacking.

Administrative systems with high degrees of formalism give executives a sense of anxiety [31]. Due to a lack of authorization, communication, and a lack of trust in supervisors, there is a high level of paperwork, mainly due to poor coordination in a highly formalistic agency [29]. Work related to pandemic control by police officers tended to increase the work pressure due to poor coordination. In addition, the lack of decision-making influence in the lower ranks of the civil service increased the work pressure of police officers in the face of the highly contagious, early-stage pandemic, the uncertainty of infection pathways, and the low level of decision-making influence [76].

Organizations with a high degree of formalism do not like change [29]. In the face of an urgent COVID-19 outbreak, traditional law enforcement and administrative processes can be stressful for frontline officers. Formalist organizations only adhere to regulations and formal procedures [76], which can lead police officers into formalistic compliance in their law enforcement. Formalistic organizations with poor administrative efficiency, confusing chains of command, and poor communication naturally increase the sense of job stress and burnout for frontline officers in an emergency outbreak [76].

**H7:**  *Perceived formalism positively influences job stress.*

**H8:**  *Perceived formalism positively influences job burnout.*

**Figure 1:**
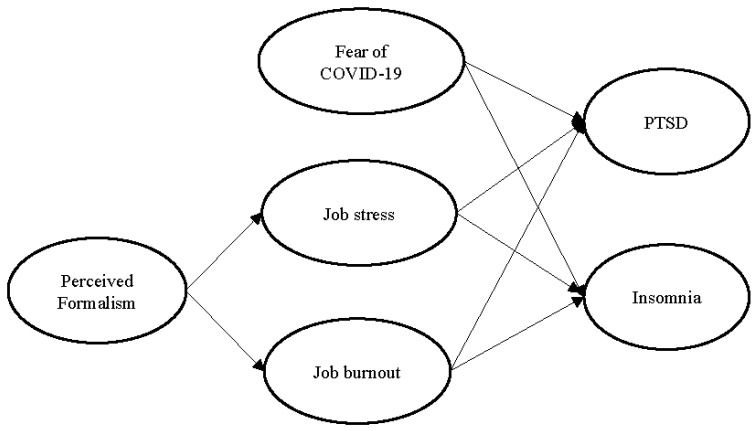
Conceptual framework.

All the above hypothesized relationships are drawn in Figure 1 below.

## 3. Materials and Methods

### 3.1. Sample, Tools, and Procedure

The questionnaire subjects of this study include police personnel across Taiwan. The questionnaire was administered from August to October 2022. At that time, the police in Taiwan were carrying out epidemic prevention work. Based on the stratification of Northern, Central, Southern, and Eastern Taiwan, questionnaire collection quotas for each region were set. Finally, 306 valid police personnel samples were obtained. This study used G*Power version 3.1.9.7 to calculate the required sample size. In this study, α err prob = 0.05, Power (1-β err prob) = 0.95, and the calculated total sample size = 132. The 306 samples we collected exceed the number of samples calculated by G*Power. Respondents were informed that this questionnaire should be answered in an anonymous and non-identifiable manner, and the research data would be stored in the host’s laboratory and would be deleted in December 2023. The research team would try its best to maintain the privacy of the respondents and fulfill its duty of confidentiality to minimize possible risks. Respondents were free to decide whether to fill out the questionnaire, and could quit at any time without feeling pressure. Regarding the basic information of the sample, 88.0 % of the respondents were male and 12.0 % were female (see Table 1). In terms of age, 13.2% were aged 20–29, 23.1% were aged 30–39, 30.0% were aged 40–49, and 33.6% were aged 50 or above. As for education level, 31.0% of respondents had received a Junior college degree, and 69.0% had at least an undergraduate degree. With respect to years of experience, 23.8% of the respondents had 10 years or less of police service, while 76.2% had 11 or more years of service. As for the marital status of the respondents, 77.0% were married and 23.0% were unmarried. Because it was not a random sample, this study conducted population and sample chi-square tests based on the statistical data of the National Police Agency in 2022. The sample data were weighted and then subjected to population and sample chi-square tests. The chi-square values of age, gender, education level, and seniority are 0.053, 0.027, 0.292, and 2.353, respectively. The significance levels of the chi-square test for age, gender, education, and seniority are 0.997, 0.869, 0.864, and 0.671, respectively, all of which do not reach the statistical significance level. It is confirmed that the sample drawn in this study is very similar to the population, and the research results can be inferred to the police population.

### 3.2. Measures

This study refers to a scale that is stable and consistent in past research and is suitable for research on police duty issues. The fear of COVID-19 scale was modified from a questionnaire designed by Ahorsu et al. [24]. Item example: I am most afraid of coronavirus-19. My hands become clammy when I think about coronavirus-19. This construct used a 5-point Likert scale, with 1 indicating strongly disagree and 5 indicating strongly agree. Cronbach’s Alpha = 0.87.

The job burnout scale was modified from a questionnaire designed by Maslach et al. [77]. Item example: I feel emotionally drained from work. I wake up exhausted every morning and have to deal with a long day at work. This construct used a 7-point Likert scale, with 1 indicating strongly disagree and 7 indicating strongly agree. Cronbach’s Alpha = 0.86.

The PTSD scale was modified from a questionnaire designed by Weathers et al. [78]. Item example: Repeated, disturbing memories, thoughts, or images caused by COVID-19 stressful experience often bothers me. Repeated, disturbing dreams often occur after COVID-19 duty. This construct used a 7-point Likert scale, with 1 indicating strongly disagree and 7 indicating strongly agree. Cronbach’s Alpha = 0.87.

The job stress scale was modified from a questionnaire designed by Cohen et al. [79]. Item example: Unexpected things often happen at work, which makes me feel frustrated. I often feel nervous and stressed while on duty. This construct used a 7-point Likert scale, with 1 indicating strongly disagree and 7 indicating strongly agree. Cronbach’s Alpha = 0.92. 

The insomnia scale was modified from a questionnaire designed by Bastien et al. [80]. Item example: I find it difficult to fall asleep. I can’t stay asleep for very long. This construct used a 7-point Likert scale, with 1 indicating strongly disagree and 7 indicating strongly agree. Cronbach’s Alpha = 0.76. 

According to the questionnaire and definition of Liu and Riggs [81,82], this study designed the following items: I think the epidemic prevention regulations and the actual implementation will not be exactly the same. I feel that the anti-epidemic laws are sometimes difficult to be fully implemented. I think many epidemic prevention systems are not easy to implement. I think there will be differences between the regulations on epidemic prevention and the status quo of implementation. This construct used a 7-point Likert scale, with 1 indicating strongly disagree and 7 indicating strongly agree. Cronbach’s Alpha = 0.94. 

### 3.3. Validity and Reliability Analysis

This study uses the R language package ‘lavaan’ for data analysis. The ‘lavaan’ package is suitable for a variety of latent variable models, including confirmatory factor analysis, structural equation modeling, and latent growth curve models. This study utilizes confirmatory factor analysis (CFA) to conduct reliability and validity testing. In terms of absolute fit measures, Chi-square = 1244, degrees of freedom = 265, and Chi-square/DF = 4.69. The goodness of fit index (GFI) is 0.98, which is higher than 0.90. The standardized root mean square residual (SRMR) is 0.12, slightly higher than 0.08. The value of RMSEA (root mean square error of approximation) is 0.11, slightly higher than the critical value of 0.10. The 95% confidence intervals of RMSEA are 0.107–0.12. In terms of model comparison fit measures, the non-normed fit index (NNFI) is 0.93, the normed fit index (NFI) is 0.93, the comparative fit index (CFI) is 0.93, the incremental fit index (IFI) is 0.94, and the relative fit index (RFI) is 0.93, all of which are higher than the critical value of 0.90. In terms of model parsimonious fit measures, the parsimonious normed fit index (PNFI) is 0.83, and the parsimonious goodness of fit index (PGFI) is 0.74, both of which are higher than the critical value of 0.50. All of the above indicators confirm the appropriateness of the conceptual model and its strong construct validity.

The factor λ values for all items range from 0.63 to 0.99, all of which are higher than 0.5. They meet the construct validity threshold recommended by Hair, Anderson, Tatham, & Black (>0.5) [83]. Additionally, the t-values for all factor λ values reach statistical significance, further confirming the construct validity and convergent validity of this study.

The main objective of assessing the composite reliability (CR) of latent variables is to measure the internal consistency of the measurement variables. A CR value must surpass 0.7 to indicate a favorable measurement quality for the latent variable [83]. The CR values in this study range from 0.76 to 0.94, all exceeding the critical threshold of 0.7 [83]. This indicates that the latent variables in this study exhibit strong internal consistency. 

Average variance extraction (AVE) represents the percentage of which a latent variable can be measured by questionnaire items. AVE is considered an indicator of reliability, convergent validity, and discriminant validity. An average variance extraction (AVE) greater than 0.5 is regarded as indicative of the convergent and discriminant validity of the research constructs [84]. In this study, the AVE values range from 0.52 to 0.80, all exceeding the threshold of 0.50. The Cronbach alpha of all latent variables ranges from 0.76 to 0.94 (Table 2), higher than the 0.70 set by Nunnally [85].

### 3.4. Inter-Correlations

When the square root of the average variance extracted (AVE) is greater than the correlation coefficient between variables, it is considered to exhibit discriminant validity [86]. The square roots of the AVE in this study range from 0.72 to 0.89, all of which are greater than the inter-construct correlation coefficients. Furthermore, the AVE values in this study are all greater than the maximum shared variance (MSV) and average shared variance (ASV), providing further evidence of the discriminant validity of the study constructs [83]. The upper-right portion of the diagonal in the correlation matrix represents the heterotrait–monotrait (HTMT) ratio of correlations. In this study, all HTMT values are below 0.90, which further confirms the discriminant validity of the study constructs [87].

As evident from the correlation coefficient matrix table, perceived formalism is positively correlated with job burnout, PTSD, job stress, and insomnia, with correlation coefficients of 0.22, 0.10, 0.13, and 0.17, respectively. It means that the higher the formalism cognition of the police is, the higher their job burnout and job stress are. The correlation coefficients of fear of COVID-19 and PTSD and insomnia are 0.48 and 0.29, respectively, indicating that the police’s fear of COVID-19 causes PTSD and insomnia problems. The correlation coefficients of job burnout and PTSD and insomnia are 0.56 and 0.62, respectively. The correlation coefficients of job stress and PTSD and insomnia are 0.63 and 0.71, respectively. It means that the job burnout and job stress of the police will increase the problems of PTSD and insomnia (see Table 3).

### 3.5. Control for Common Method Variance

Common method variance (CMV) is a variation caused by a measurement method that will cause an internal consistency error [88,89]. This study used a self-administered questionnaire that was relatively prone to CMV problems. In order to avoid CMV problems, the questionnaire in this study was administered in an anonymous manner, using a mixed Likert scale of 5–7 points [88]. In addition, the questionnaire was designed to be simple and easy to understand to avoid confusion, misunderstanding, and difficulty for respondents. 

In this study, Harman’s one-factor test was used for the post hoc test of CMV. The exploratory factor analysis explained 39.4% of the variance of the first factor in the state of no rotation axis. Such results confirm that this study is relatively immune to CMV errors.

## 4. Results and Data Analysis

In this study, path coefficient analysis of structural equation modeling (SEM) was used to test the established hypotheses. Chi-square = 1244, degrees of freedom = 265, Chi-square/DF = 4.69. The value of RMSEA (root mean square error of approximation) is 0.11, slightly higher than the critical value of 0.10. The 95% confidence intervals of RMSEA are 0.107–0.12. The goodness of fit index (GFI), non-normed fit index (NNFI), normed fit index (NFI), comparative fit index (CFI), incremental fit index (IFI) and the relative fit index (RFI) are all higher than the critical value of 0.90. Table 4 shows that job stress positively affects PTSD, with a standardized coefficient of 0.53. This is statistically significant, and leads to acceptance of H1. PTSD can be triggered when police officers experience high levels of job stress. Police work is inherently characterized by many work-related stressors. In the case of the COVID-19 outbreak, the uncertainty of policy, a lack of effective vaccines, and insufficient quantities of protective equipment have made police officers more susceptible to developing PTSD as a result of increased work pressure. The loss of social support has also made the situation difficult for the police officers.

Fear of COVID-19 worsens PTSD in police officers, with a standardized coefficient of 0.31 and a statistically significant acceptance of H2. Fear of COVID-19 is a construct that has developed during the outbreak. Police officers working on the front lines of law enforcement during the outbreak have often been anxious and fearful of being infected. The pandemic has lasted 2–3 years and has caused high levels of fear in police officers. PTSD also occurred in frontline officers during the MERS and SARS outbreaks [41]. The stresses associated with the COVID-19 pandemic, including fear of infection, fear of death, financial hardship, and the disruption to their lives, have made police officers susceptible to PTSD [51].

This study demonstrates that job burnout positively affects PTSD, with a standardized coefficient of 0.13 (*p* < 0.001), leading to acceptance of H3. Police officers had not previously been exposed to COVID-19 outbreak duty-related training, and the rapid spread of the outbreak has predisposed them to job burnout. The frustration, anger, and exhaustion associated with job burnout further contributes to their PTSD [55]. Police officers are subjected to more work demands during an outbreak, predisposing them to higher levels of both job burnout and PTSD [58]. The ongoing outbreak has lasted 2–3 years, predisposing police officers to being caught up in ruminating over traumatic events, which in turn has contributed to the development of PTSD.

Job stress positively affects insomnia, with a standardized coefficient of 0.66 (*p* < 0.001), leading to acceptance of H4. Uncertainty, worry, and anxiety associated with a pandemic can easily cause insomnia in frontline officers [66]. Insomnia induced by stressful events can be intermittent, recurrent, and chronic [63], which can be detrimental to officers’ physical and mental health. When police officers have excessive workloads due to the pandemic; isolation from family, relatives and friends; lack of adequate support; and even discrimination [46], these can all contribute to their increased work stress. Insomnia has been recognized as one of the most important symptoms of stress. Past studies have found that insomnia is an underestimated and underdiagnosed symptom [63]. Insomnia among police officers due to pandemic stress should be taken seriously.

Fear of COVID-19 positively affects insomnia, with a standardized coefficient of 0.06 (*p* < 0.001), leading to acceptance of H5. Fear of COVID-19 is thought to cause anxiety and insomnia problems [19]. COVID-19 is life-threatening in a way that has not occurred before, and the sense of fear experienced on the job can easily lead to insomnia [6,40]. Lack of protective equipment and vaccines can cause fear and resultant insomnia problems in police officers.

Job burnout positively affects insomnia, with a standardized coefficient of 0.23 (*p* < 0.001), leading to acceptance of H6. The effects of job burnout encompass emotional exhaustion, depersonalization, and diminished feelings of personal accomplishment [20], which predispose officers to insomnia. The lockdown and social distancing policies that police officers have faced during the pandemic have resulted in increased workloads and shift changes, all contributing to job burnout and resultant insomnia. Past studies have also confirmed that job burnout is recognized as a cause of insomnia [75].

Police officers’ perceived formalism positively affects job stress, with a standardized coefficient of 0.87 (*p* < 0.001), leading to acceptance of H7. Police officers with a high degree of formalism perception will perceive that there is a discrepancy between anti-pandemic policies and actual duty. In the absence of authorization and communication, and with mistrust of supervisors, it is easy to develop a sense of anxiety. Especially when anti-pandemic training is insufficient, police officers are prone to job burnout [31]. The lack of authorization, overemphasis on regulations, documentation, and lack of communication in a highly formalistic organization prevent officers from being able to enforce the law to the fullest extent [31]. Poor agency coordination and a high level of paperwork also cause police officers to spend a lot of time dealing with internal pressures [29]. Formalistic agencies that only follow regulations and formal procedures can increase job stress among frontline officers [76].

Perceived formalism positively affects job burnout, with a standardized coefficient of 0.91 (*p* < 0.001), leading to acceptance of H8. Organizations with a high degree of formalism are averse to change [29], and feel pressured to adapt to new situations and tasks. Formalist organizations emphasize adherence to past regulations and formal procedures [76], and have difficulty adapting to the enforcement of new laws and regulations arising from a pandemic. In addition, formalistic organizations are characterized by poor administrative efficiency and poor communication [76], which can create a sense of burnout for frontline law enforcement officers.

## 5. Conclusions

This study first confirms that police officers’ perceived formalism positively affects job stress. If the police believe that epidemic prevention laws cannot be implemented, it will increase their work pressure. In the past, most of the research on formalism was conducted from a qualitative perspective, and the impact of formalism on the cognition of frontline personnel was rarely confirmed from an empirical perspective. This study also confirms that perceived formalism positively affects job burnout. When police officers feel that there is a discrepancy between laws and actual implementation, they will feel anxious and fatigued about epidemic prevention work. Poor communication in formalistic organizations also increases the police’s job burnout [76].

Police personnel’s job stress positively affects PTSD. The uncertainty and lack of equipment caused by the COVID-19 epidemic have caused PTSD among police officers. This study also confirms that the job stress of police officers positively affects insomnia. The COVID-19 epidemic’s high contagiousness, high mortality rate, and work overload have increased the work pressure of police officers [46]. These work stressors further cause police officers to suffer from insomnia. 

Fear of COVID-19 is the scale that has developed during the pandemic. This study confirms that high levels of police fear of COVID-19 can cause PTSD. The fear caused by the high contagion and mortality rate of COVID-19 has increased police officers’ PTSD [51]. This study also confirms that the police’s fear of COVID-19 positively affects insomnia. The lack of medicine and the fear of high mortality make police officers working on the front line prone to insomnia [6,40].

This study confirms that police job burnout positively affects PTSD. The anxiety, exhaustion, and burnout of police work can easily lead to PTSD [55]. The emotional exhaustion, depersonalization, and diminished feelings of personal accomplishment of police work also make police officers prone to insomnia [20]. Shift changes and increased workload caused by the COVID-19 epidemic have increased police burnout and insomnia problems.

## 6. Theoretical and Practical Implications

In terms of theoretical contribution, this study attempts to correct the problem of insufficient empirical research on formalism in administrative management. The formalism construct is introduced into the attitudinal cognition of police officers in anti-pandemic and law enforcement work, to explore its effects on job burnout and work stress. In the past, most of the literature on formalism has been characterized by qualitative and descriptive statistics. In this study, an inferential statistical perspective was used to explore the impact of formalism on administrative agencies.

This study has found that formalism has increased the work stress and burnout among police officers on duty during the pandemic, resulting in insomnia and PTSD. The main problem of formalism stems from a lack of an objective evaluation mechanism in administrative agencies. The lack of an assessment mechanism eliminates the need for civil servants and police officers to work hard on their performance. As a result, there is an inconsistency between administrative agencies’ requirements and officers’ actual performance in implementation. During the pandemic, poor communication and a focus on administrative procedures have made it difficult for frontline police officers to perform their duties, resulting in higher work pressure and burnout. Taiwan’s civil servants’ performance appraisal lacks objective standards; a considerable proportion of civil servants in Taiwan’s government agencies even just take turns obtaining performance evaluations of “A” and “B”. Police agencies should establish clear performance evaluation standards to reduce the generation of law enforcement formalism.

This study confirms that fear of COVID-19 positively affects PTSD and insomnia in police officers. This study suggests that police agencies should understand the causes of anxiety and fear in order to manage the fear caused by a virus. Credible, accurate information should be provided to police officers during an outbreak, to avoid social media misinformation resulting in panic on duty. When infected on duty, the focus should be on manageable options, including staying away from crowds, getting enough sleep, and enforcing social distancing policies. Police agencies and superintendents should provide administrative and leadership support. Many social supports have been disrupted during the outbreak, coupled with increasing work demands. Social support can reduce the stress and burnout associated with job demands.

Police officers’ work stress also positively affects PTSD and insomnia. Police authorities and supervisors should identify sources of work stress among police officers during the pandemic and provide appropriate social support. Unknown viral infection pathways, a rapidly-spreading virus, and the implementation of quarantine policies all contribute to work stress, PTSD, and insomnia among police officers. Transformational leadership is more appropriate for police management during an outbreak.

Job burnout among police officers positively affects PTSD and insomnia. Being on duty during a pandemic can easily lead to conflict with coworkers and members of the public. Police officers who are particularly formalistic may perceive there to be administrative inefficiencies and poor communication, easily leading to conflicts with supervisors and coworkers, and thus resulting in burnout. Police officers should manage job burnout appropriately and seek support from their organizations and colleagues. Police authorities or supervisors should help police officers break down multiple pandemic tasks into smaller tasks and accomplish them separately.

## 7. Research Limitations and Future Research Suggestions

Due to limited time and financial resources in this study, the sample of this study may suffer from “sample bias” or “selection bias”. Although the 306 samples in this paper exceed the number of samples calculated by G*Power, in order to generalize the research results, it is recommended that future researchers collect more samples for in-depth analysis. The samples collected in Taiwan may be affected by the cultural background. It is recommended that future researchers collect samples from various countries for comparative analysis. Quantitative research adopts a closed questionnaire, which sometimes limits the answers of the respondents. It is suggested that future researchers adopt qualitative analysis for more in-depth discussion. The Chi-square/df and RMSEA of this study are somewhat beyond the generally accepted levels. This demonstrates that other relationships between latent factors in this research framework may exist. Policy formalism is a factor that the authors have tried to explore in recent years to supplement the lack of empirical data in formalism research. In the early stage of formalism research, it was not possible to find much literature supporting the relationship between formalism and other latent factors. Future researchers can use a broader theoretical perspective and try to explore unverified relationships with more in-depth literature. New hypothesis relationship testing can inject more inspiration and discovery into formalism research.

## Figures and Tables

**Table 1:** Sample basic information.

	Percentage (%)		Percentage (%)
Gender		Seniority	
Male	88.0 %	1 to 5 years	9.9%
Female	12.0 %	6 to 10 years	13.9%
Age		11 to 15 years	13.9%
20–29 years old	13.2 %	16 to 20 years	8.3%
30–39 years old	23.1 %	21 years or more	54.0%
40–49 years old	30.0 %	Marriage	
50–59 years old or older	33.6 %	Unmarried	23.0%
Education level		Married	77.0%
Junior college	31.0 %		
College	51.8 %		
Postgraduate	17.2 %		

**Table 2:** Item loading and reliability.

Variables	Items	Lambda Loading	Z Value	CR	Cronbachs Alpha
Fear of COVID-19	FC1	0.83		0.87	0.87
	FC2	0.88	42.5		
	FC3	0.80	42.3		
Job burnout	JB1	0.69		0.86	0.86
	JB2	0.63	52.5		
	JB3	0.82	59.4		
	JB4	0.75	56.9		
	JB5	0.79	57.7		
PTSD	PTSD1	0.80		0.87	0.87
	PTSD2	0.77	63.5		
	PTSD3	0.69	59.3		
	PTSD4	0.69	60.3		
	PTSD5	0.82	63.3		
Job stress	JS1	0.87		0.92	0.92
	JS2	0.84	69.5		
	JS3	0.85	71.9		
	JS4	0.78	70.9		
	JS5	0.84	70.0		
Insomnia	IS1	0.77		0.76	0.76
	IS2	0.73	59.1		
	IS3	0.67	55.9		
Perceived formalism	PF1	0.80		0.94	0.94
	PF2	0.87	24.9		
	PF3	0.99	25.6		
	PF4	0.88	24.8		

Note: FC = fear of COVID-19; JB = job burnout; JS = job stress; IS = insomnia; PF = perceived formalism. Please see Appendix A Table A1 for detailed items. The first item of each variable is fixed to 1, so there is no Z value.

**Table 3:** Square root of AVE and inter-correlations.

	1	2	3	4	5	6	ASV	MSV	AVE
Perceived formalism (1)	(0.89)	0.04	0.25	0.13	0.15	0.19	0.02	0.05	0.80
Fear of COVID-19 (2)	−0.07	(0.83)	0.30	0.50	0.36	0.23	0.11	0.23	0.69
Job burnout (3)	0.22	0.31	(0.74)	0.65	0.79	0.78	0.27	0.51	0.55
PTSD (4)	0.10	0.48	0.56	(0.76)	0.73	0.78	0.26	0.39	0.57
Job stress (5)	0.13	0.35	0.71	0.63	(0.84)	0.88	0.31	0.51	0.70
Insomnia (6)	0.17	0.29	0.62	0.59	0.71	(0.72)	0.27	0.51	0.52

Note: The figures in parentheses indicate the square root of AVE of the study constructs. The lower left table on the diagonal is the Pearson correlation coefficient, and the upper right table is the heterotrait–monotrait (HTMT) ratio of correlations. MSV = maximum share variance, ASV = average share variance.

**Table 4:** Path coefficients.

	Causal Path			Path Coefficient	Standard Error	Z Value	*p* Value
H1	Job stress	->	PTSD	0.53	0.03	15.75	<0.001
H2	Fear of COVID-19	->	PTSD	0.31	0.01	21.52	<0.001
H3	Job burnout	->	PTSD	0.13	0.04	3.85	<0.001
H4	Job stress	->	Insomnia	0.66	0.04	15.37	< 0.001
H5	Fear of COVID-19	->	Insomnia	0.06	0.01	3.88	<0.001
H6	Job burnout		Insomnia	0.23	0.05	5.32	<0.001
H7	Perceived formalism	->	Job stress	0.87	0.21	20.02	< 0.001
H8	Perceived formalism	->	Job burnout	0.91	0.16	20.54	< 0.001

## Data Availability

Data are unavailable due to privacy or ethical restrictions.

## Appendix A

**Table A1:** Variables, items and descriptive statistics tables.

Variables	Items	Mean	Standard Deviation
Fear of COVID-19	I am most afraid of coronavirus-19	3.14	1.84
	My hands become clammy when I think about coronavirus-19	2.73	1.57
	I am afraid of losing my life because of coronavirus-19	3.09	1.68
Job burnout	I feel emotionally drained from work	4.72	1.57
	I wake up exhausted every morning and have to deal with a long day at work	4.61	1.57
	I feel depleted after work every day.	4.17	1.65
	I’m worried that working during the epidemic will worsen my mood	4.68	1.59
	I’m worried that people will blame me for my duty due to the epidemic	3.31	1.58
PTSD	Repeated, disturbing memories, thoughts, or images caused by COVID-19 stressful experience often bothers me	3.41	1.63
	Repeated, disturbing dreams often occur after COVID-19 duty	3.51	1.64
	I get frustrated when anyone brings up the events surrounding COVID-19	3.35	1.59
	I started to lose interest in the things I used to enjoy	3.60	1.63
	When I think about what happened during the epidemic, my heart is pounding and trouble breathing, sweating	3.38	1.54
Job stress	Unexpected things often happen at work, which makes me feel frustrated	3.98	1.65
	I often feel nervous and stressed while on duty	3.63	1.54
	While on duty, I found that I could not fully deal with the problems I faced	3.44	1.66
	I often feel like there is still a lot of work to be done	3.52	1.73
	There are many difficulties that are not easy to solve in the process of duty	3.13	1.57
Insomnia	I find it difficult to fall asleep	3.15	1.53
	I can’t stay asleep for very long	3.68	1.70
	I have a problem waking up early	4.57	1.63
Perceived formalism	I think the epidemic prevention regulations and the actual implementation will not be exactly the same	5.30	1.29
	I feel that the anti-epidemic laws are sometimes difficult to be fully implemented	5.48	1.18
	I think many epidemic prevention systems are not easy to implement	5.32	1.23
	I think there will be differences between the regulations on epidemic prevention and the status quo of implementation	5.44	1.16
